# Response to “Susceptibility to diabetic nephropathy”

**DOI:** 10.1007/s00467-024-06298-5

**Published:** 2024-01-19

**Authors:** Alexander J. Howie, David V. Milford

**Affiliations:** 1 Department of Histopathology, Birmingham Children’s Hospital, Birmingham, B4 6NH UK; 2 Department of Nephrology, Birmingham Children’s Hospital, Birmingham, B4 6NH UK

We thank Dr. Nubuo Tsuboi [1] for interest in our paper [2]. Unfortunately, much of the information that Dr. Tsuboi requests is unavailable, either because the clinical notes especially from the early years of our study were incomplete, or because requested details were not investigated. With so few children in each of our groups, comparisons between the groups could only be suggestive rather than definitive evidence of susceptibility to diabetic nephropathy. In relation to the duration of diabetes, the three children with diabetic nephropathy and IgA nephropathy had a shorter median duration of diabetes, 79 months, than the four with diabetic nephropathy on its own, 116 months, and both these groups had a longer median duration than the ten with kidney disorders other than diabetic nephropathy, 72 months. With such small groups, firm statistical conclusions are impossible, and in any event, interpretation of susceptibility would need thorough comparisons with at least a sample of the whole population of diabetic children, details of which were not available to us.
